# Phylogenetic Insights into the Functional Relationship between Primate Lentiviral Reverse Transcriptase and Accessory Proteins Vpx/Vpr

**DOI:** 10.3389/fmicb.2016.01655

**Published:** 2016-10-18

**Authors:** Yosuke Sakai, Naoya Doi, Yasuyuki Miyazaki, Akio Adachi, Masako Nomaguchi

**Affiliations:** ^1^Department of Microbiology, Tokushima University Graduate School of Medical ScienceTokushima, Japan; ^2^Department of Microbiology and Cell Biology, Tokyo Metropolitan Institute of Medical ScienceTokyo, Japan

**Keywords:** HIV, SIV, Vpx, Vpr, RT, SAMHD1, dNTP

## Abstract

The efficiency of reverse transcription to synthesize viral DNA in infected cells greatly influences replication kinetics of retroviruses. However, viral replication in non-dividing cells such as resting T cells and terminally differentiated macrophages is potently and kinetically restricted by a host antiviral factor designated SAMHD1 (sterile alpha motif and HD-domain containing protein 1). SAMHD1 reduces cellular deoxynucleoside triphosphate (dNTP) pools and affects viral reverse transcription step. Human immunodeficiency virus type 2 (HIV-2) and some simian immunodeficiency viruses (SIVs) have Vpx or Vpr to efficiently degrade SAMHD1. Interestingly, the reverse transcriptase (RT) derived from HIV-1 that encodes no anti-SAMHD1 proteins has been previously demonstrated to uniquely exhibit a high enzymatic activity. It is thus not irrational to assume that some viruses may have acquired or lost the specific RT property to better adapt themselves to the low dNTP environments confronted in non-dividing cells. This adaptation process may probably be correlated with the SAMHD1-antagonizing ability by viruses. In this report, we asked whether such adaptive events can be inferable from Vpx/Vpr and RT phylogenetic trees overlaid with SAMHD1-degrading capacity of Vpx/Vpr and with kinetic characteristics of RT. Resultant two trees showed substantially similar clustering patterns, and therefore suggested that the properties of RT and Vpx/Vpr can be linked. In other words, HIV/SIVs may possess their own RT proteins to adequately react to various dNTP circumstances in target cells.

## Introduction

Accessory proteins of various human immunodeficiency viruses/simian immunodeficiency viruses (HIV/SIVs) are believed to be essential for optimal viral replication, persistence, and pathogenicity *in vivo* (Matheson et al., 2016). Vpx was least well studied among the five accessory proteins (Vif, Vpx, Vpr, Vpu, and Nef) for its functional role and the underlying molecular basis until the recent identification of cellular anti-viral factor SAMHD1 as a target for Vpx (Hrecka et al., 2011; Laguette et al., 2011). Extensive studies since then have generated a series of critical findings to understand the interaction of Vpx and SAMHD1 (Goldstone et al., 2011; Hrecka et al., 2011; Laguette et al., 2011, 2012; Baldauf et al., 2012; Descours et al., 2012; Lahouassa et al., 2012; Lim et al., 2012; St Gelais et al., 2012; Lenzi et al., 2014, 2015): (1) all Vpx and some Vpr proteins derived from various HIV/SIVs target SAMHD1 for proteasomal degradation; (2) SAMHD1 reduces cellular deoxynucleoside triphosphate (dNTP) pools to a level similar to that observed in non-dividing myeloid and resting T cells; (3) HIV-1 reverse transcriptase (RT) shows a high binding affinity to dNTPs relative to those from other lentiviruses. Of note here, HIV-1 can replicate in macrophages to some extent, and lacks SAMHD1-degrading activity. Thus, HIV-1 appears to be unique among primate lentiviruses to act against SAMHD1.

On the basis of the experimental results summarized above, it would hold true to hypothesize that some specific viruses have adapted themselves to better fit the physiological environments in non-dividing cells. Therefore, we here have performed phylogenetic analyses using distinct Vpx/Vpr and RT proteins from viruses with/without anti-SAMHD1 activity. To this end, we phylogenetically examined the target viral proteins (Vpx/Vpr and RT with no sequence ambiguity) derived from three types of diverse SIVs based on their genome features relating to *vpx, vpr*, and *vpu* genes (Fujita et al., 2010; Sakai et al., 2016) as follows. (1) “Prototype viruses” carrying *vpr* gene analyzed here were SIVagm (isolated from the African green monkey), SIVmnd-1 (mandrill), SIVlst (l’Hoest’s monkey), SIVsun (sun-tailed monkey), SIVsyk (Sykes’ monkey), SIVdeb (DeBrazza’s monkey), SIVtal (talapoin monkey), SIVasc (red-tailed guenon), SIVcol (colobus monkey), SIVwrc (western red colobus), and SIVolc (olive colobus). (2) “HIV-1 type viruses” carrying *vpr* and *vpu* genes analyzed here were, SIVcpz (chimpanzee), SIVgor (gorilla), SIVgsn (greater spot-nosed monkey), SIVmon (mona monkey), SIVmus (mustached monkey), and SIVden (Dent’s monkey). (3) “HIV-2 type viruses” carrying *vpx* and *vpr* genes analyzed here were SIVsmm (sooty mangabey monkey), SIVmac (macaque monkey), SIVmne (pig-tailed macaque), SIVstm (stump-tailed macaque), SIVrcm (red-capped mangabey), SIVmnd-2 (mandrill), and SIVdrl (drill monkey).

## Phylogeny of Vpx/Vpr Proteins Derived From Viruses With/Without Samhd1-Degrading Activity

Early studies have shown that Vpx is essential for HIV-2 and SIVmac replication in primary non-dividing cells (Fujita et al., 2010; Schaller et al., 2014). Subsequent studies have revealed that Vpx enhances viral DNA synthesis by inducing proteasomal degradation of an anti-viral factor in those cells (Fujita et al., 2010; Schaller et al., 2014). It is now established that the anti-viral factor SAMHD1 abundantly present in the non-dividing cells is degraded by Vpx from HIV-2 type viruses and Vpr from some prototype viruses (Laguette et al., 2012; Lim et al., 2012). There are no Vpr proteins with SAMHD1-degrading activity derived from HIV-1 and HIV-2 type viruses except for Vpr from SIVmus (HIV-1 type) (Laguette et al., 2012; Lim et al., 2012).

**FIGURE 1:**
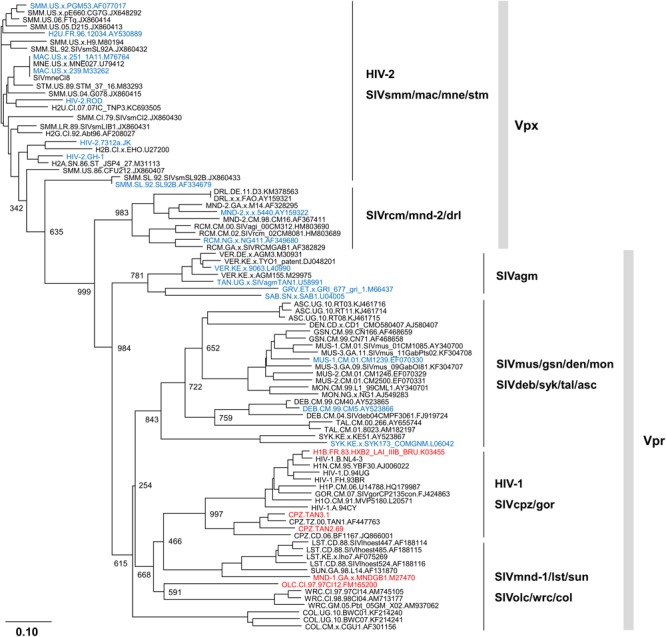
**Phylogeny of Vpx/Vpr proteins derived from various human immunodeficiency viruses/simian immunodeficiency viruses (HIV/SIVs).** Phylogenetic tree based on diverse Vpx/Vpr proteins was constructed as previously described (Sakai et al., 2016), and is shown together with SAMHD1 (sterile alpha motif and HD-domain containing protein 1)-degrading activity. Viral clones with/without SAMHD1-degrading activity (Laguette et al., 2012; Lim et al., 2012) are indicated by blue and red letters, respectively. Note that Vpr proteins from HIV-2 type viruses (HIV-2, SIVsmm/mac/mne/stm, and SIVrcm/mnd-2/drl) are excluded from this tree for simplicity and clarity. Branches were calculated from 1000 bootstrap replicates, and the bootstrap values are labeled on the major branches. Scale bar represents the genetic distance. For virus designations, origins, and types, see the text (Introduction). Amino acid sequences were obtained from the HIV sequence database at Los Alamos National Laboratory (http://www.hiv.lanl.gov) or from the GenBank (http://www.ncbi.nlm.nih.gov).

In order to easily see the genetic background for the results described above (Laguette et al., 2012; Lim et al., 2012), we also inferred a bootstrap phylogenetic tree of Vpx/Vpr proteins from diverse HIV/SIVs by the neighbor-joining method as previously described (Sakai et al., 2016), and the proteins with/without SAMHD1-degrading activity were highlighted by blue and red letters, respectively. Viruses without Vpx (prototype and HIV-1 type viruses) were analyzed for their Vpr proteins, and viruses with both Vpx and Vpr (HIV-2 type viruses) were examined for their Vpx proteins. As shown in **Figure 1**, Vpx/Vpr proteins from viruses with SAMHD1-degrading activity (viral groups: HIV-2, SIVsmm/mac/mne/stm, SIVrcm/mnd-2/drl, SIVagm, SIVmus/gsn/den/mon, and SIVdeb/syk/tal/asc) clearly formed different clusters from those by Vpr proteins from viruses without SAMHD1-degrading activity (viral groups: HIV-1, SIVcpz/gor, SIVmnd-1/lst/sun, and SIVolc/wrc/col). No virus strains without SAMHD1-degrading activity were found in the clusters with the activity and the opposite was true. These results suggested that HIV/SIVs with/without anti-SAMHD1 activity diverged at some time point in the past.

## Phylogeny of Rt Proteins Derived From Viruses With/Without Samhd1-Degrading Activity

Human immunodeficiency virus type 1 does not encode any anti-SAMHD1 proteins as described above, which appears to be disadvantageous to the virus for efficient replication in target cells, especially in non-dividing myeloid and resting T cells. Nonetheless, HIV-1 grows well in humans and is markedly pathogenic for humans. While the mechanism(s) underlying for this viral property remains elusive, one could postulate, as a likely possibility, that the high growth ability of HIV-1 is attributable to its relatively high RT activity (Schaller et al., 2014). Indeed, it has been reported that HIV-1 RT is more active in synthesizing viral DNA than the other viral RT proteins derived from viruses with SAMHD1-degrading ability (Lenzi et al., 2014, 2015).

**FIGURE 2:**
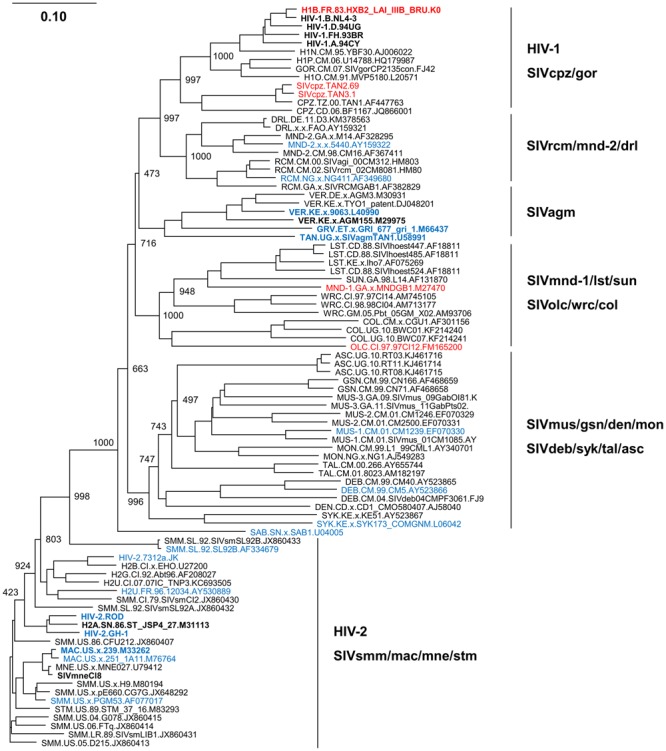
**Phylogeny of reverse transcriptase (RT) proteins derived from various HIV/SIVs.** Phylogenetic tree based on diverse RT proteins was constructed as previously described (Sakai et al., 2016), and is shown together with SAMHD1-degrading and RT activities. Viral clones with/without SAMHD1-degrading activity (Laguette et al., 2012; Lim et al., 2012) are indicated by blue and red letters, respectively. In addition, viral clones experimentally tested for RT activity (Lenzi et al., 2014, 2015) are indicated by boldface type letters. Note that RT proteins from the HIV-1 group show a higher enzymatic activity relative to those from the other viral groups examined (Lenzi et al., 2014, 2015). Branches were calculated from 1000 bootstrap replicates, and the bootstrap values are labeled on the major branches. Scale bar represents the genetic distance. For virus designations, origins, and types, see the text (Introduction). Amino acid sequences were obtained from the HIV sequence database at Los Alamos National Laboratory (http://www.hiv.lanl.gov) or from the GenBank (http://www.ncbi.nlm.nih.gov).

We therefore constructed a phylogenetic tree of various RT proteins from viruses with/without anti-SAMHD1 activity in order to predict their evolutional positions as described above (Sakai et al., 2016), and the result was shown in **Figure 2**. RT proteins from viruses without anti-SAMHD1 activity (viral groups: HIV-1, SIVcpz/gor, SIVmnd-1/lst/sun, and SIVolc/wrc/col) and those from viruses with the activity (viral groups: SIVrcm/mnd-2/drl, SIVagm, SIVmus/gsn/den/mon, SIVdeb/syk/tal/asc, HIV-2, and SIVsmm/mac/mne/stm) separately formed clusters as observed in the Vpx/Vpr tree (**Figure 2**). Importantly, viruses with a high RT activity (Lenzi et al., 2014, 2015) were confined to the HIV-1 cluster without SAMHD1-degrading ability. In contrast, viruses with a low RT activity (Lenzi et al., 2014, 2015) were found in various virus clusters with SAMHD1-degrading ability (SIVagm, HIV-2, SIVmac, and SIVmne). While not included in this phylogenetic tree due to the sequence unavailability to us, two more HIV-1 strains (92RW and 93IN) and two other HIV-2/SIVmne strains (Rod10 and 170) were reported to have RT proteins with a high and low enzymatic activity, respectively (Lenzi et al., 2014). Together with the result in **Figure 1**, our phylogenetic tree here is consistent with a hypothesis that the ability of Vpx/Vpr proteins from various HIV/SIVs to degrade SAMHD1 and the different enzymatic activity of RT proteins from various HIV/SIVs are intimately linked.

## Concluding Remarks

Primate lentiviral diversification is frequently accompanied by functional alterations of viral proteins required for efficient viral replication in different cellular environments, which can provide virologically essential informations to understand the inter-/intra-species transmission and adaptation processes. In this report, we have described a potential link between the SAMHD1-degrading activity of Vpx/Vpr proteins and the enzymatic activity of RT proteins. Our phylogenetic analyses (**Figures 1** and **2**) are consistent with a scenario that HIV-1 without anti-SAMHD1 activity may already have or have acquired RT with enhanced enzymatic activity. However, because viral strains experimentally examined for their anti-SAMHD1 and RT activities through biochemical and molecular biological approaches are limited so far, it would be too early to draw a clear conclusion on this issue. Extensive experimental studies are required to obtain decisive results.

## Author Contributions

YS, AA, and MN designed the research. YS performed the phylogenetic analysis. YS, ND, YM, AA, and MN analyzed and discussed the results. YS, AA, and MN wrote the manuscript.

## Conflict of Interest Statement

The authors declare that the research was conducted in the absence of any commercial or financial relationships that could be construed as a potential conflict of interest. The reviewer AK and handling Editor declared their shared affiliation, and the handling Editor states that the process nevertheless met the standards of a fair and objective review.

## References

[B1] Baldauf H. M. Pan X. Erikson E. Schmidt S. Daddacha W. Burggraf M. (2012). SAMHD1 restricts HIV-1 infection in resting CD4+ T cells. *Nat. Med.* 18 1682–1687. 10.1038/nm.2964 22972397 PMC3828732

[B2] Descours B. Cribier A. Chable-Bessia C. Ayinde D. Rice G. Crow Y. (2012). SAMHD1 restricts HIV-1 reverse transcription in quiescent CD4(+) T-cells. *Retrovirology* 9:87 10.1186/1742-4690-9-87 PMC3494655 23092122

[B3] Fujita M. Otsuka M. Nomaguchi M. Adachi A. (2010). Multifaceted activity of HIV Vpr/Vpx proteins: the current view of their virological functions. *Rev. Med. Virol.* 20 68–76. 10.1002/rmv.636 20069611

[B4] Goldstone D. Ennis-Adeniran V. Hedden J. Groom H. Rice G. Christodoulou E. (2011). HIV-1 restriction factor SAMHD1 is a deoxynucleoside triphosphate triphosphohydrolase. *Nature* 480 379–382. 10.1038/nature10623 22056990

[B5] Hrecka K. Hao C. Gierszewska M. Swanson S. K. Kesik-Brodacka M. Srivastava S. (2011). Vpx relieves inhibition of HIV-1 infection of macrophages mediated by the SAMHD1 protein. *Nature* 474 658–661. 10.1038/nature10195 21720370 PMC3179858

[B6] Laguette N. Rahm N. Sobhian B. Chable-Bessia C. Münch J. Snoeck J. (2012). Evolutionary and functional analyses of the interaction between the myeloid restriction factor SAMHD1 and the lentiviral Vpx protein. *Cell Host Microbe* 11 205–217. 10.1016/j.chom.2012.01.007 22305291 PMC3595996

[B7] Laguette N. Sobhian B. Casartelli N. Ringeard M. Chable-Bessia C. Ségéral E. (2011). SAMHD1 is the dendritic- and myeloid-cell-specific HIV-1 restriction factor counteracted by Vpx. *Nature* 474 654–657. 10.1038/nature10117 21613998 PMC3595993

[B8] Lahouassa H. Daddacha W. Hofmann H. Ayinde D. Logue E. Dragin L. (2012). SAMHD1 restricts the replication of human immunodeficiency virus type 1 by depleting the intracellular pool of deoxynucleoside triphosphates. *Nat. Immunol.* 13 223–228. 10.1038/ni.2236 22327569 PMC3771401

[B9] Lenzi G. Domaoal R. Kim D.-H. Schinazi R. Kim B. (2015). Mechanistic and kinetic differences between reverse transcriptases of Vpx coding and non-coding lentiviruses. *J. Biol. Chem.* 290 30078–30086. 10.1074/jbc.M115.691576 26483545 PMC4705996

[B10] Lenzi G. M. Domaoal R. A. Kim D.-H. Schinazi R. F. Kim B. (2014). Kinetic variations between reverse transcriptases of viral protein X coding and noncoding lentiviruses. *Retrovirology* 11:111 10.1186/s12977-014-0111-y PMC4282736 25524560

[B11] Lim E. S. Fregoso O. I. McCoy C. O. Matsen F. A. Malik H. S. Emerman M. (2012). The ability of primate lentiviruses to degrade the monocyte restriction factor SAMHD1 preceded the birth of the viral accessory protein Vpx. *Cell Host Microbe* 11 194–204. 10.1016/j.chom.2012.01.004 22284954 PMC3288607

[B12] Matheson N. J. Greenwood E. J. D. Lehner P. J. (2016). Manipulation of immunometabolism by HIV – accessories to the crime? *Curr. Opin. Virol.* 19 65–70. 10.1016/j.coviro.2016.06.014 27448768

[B13] Sakai Y. Miyake A. Doi N. Sasada H. Miyazaki Y. Adachi A. (2016). Expression profiles of Vpx/Vpr proteins are co-related with the primate lentiviral lineage. *Front. Microbiol.* 7:1211 10.3389/fmicb.2016.01211 PMC4971069 27536295

[B14] Schaller T. Bauby H. Hué S. Malim M. H. Goujon C. (2014). New insights into an X-traordinary viral protein. *Front. Microbiol.* 5:126 10.3389/fmicb.2014.00126 PMC3986551 24782834

[B15] St Gelais C. De Silva S. Amie S. M. Coleman C. M. Hoy H. Hollenbaugh J. A. (2012). SAMHD1 restricts HIV-1 infection in dendritic cells (DCs) by dNTP depletion, but its expression in DCs and primary CD4+ T-lymphocytes cannot be upregulated by interferons. *Retrovirology* 9:105 10.1186/1742-4690-9-105 PMC3527137 23231760

